# Occurrence and Risk Factors of *Toxoplasma gondii* Infection in Tigers (*Panthera tigris*) in Thailand: Influence of Age and Sex

**DOI:** 10.1002/vms3.71114

**Published:** 2026-07-20

**Authors:** Phirom Prompiram, Chalisa Mongkolphan, Aeknarin Saechin, Siriporn Tangsudjai, Kanaporn Poltep

**Affiliations:** ^1^ Monitoring and Surveillance Center for Zoonotic Diseases in Wildlife and Exotic Animals Faculty of Veterinary Science Mahidol University Nakhon Pathom Thailand

**Keywords:** occurrence, risk factors, tigers, *Toxoplasma gondii*, zoonotic parasite

## Abstract

**Background:**

*Toxoplasma gondii* is a zoonotic protozoan parasite of significant concern in both public and animal health.

**Objectives:**

This study investigated the occurrence of *T. gondii* in 135 captive tigers (*Panthera tigris*) across seven tiger parks in Thailand. Tiger parks, as theme‐based wildlife attractions, are high‐risk venues for zoonotic disease transmission due to the close contact between humans and captive wild animals.

**Methods:**

The presence of *T. gondii* specific antibodies was determined using a latex agglutination test.

**Results:**

Serological analysis revealed that 42.96% (58/135; 95% CI: 34.48–51.76) of the tigers tested positive for *T. gondii* antibodies. Females exhibited a significantly higher occurrence of 51.43% (36/70; 39.17–63.56) compared to males at 33.85% (22/65; 22.57–46.65) (*p* = 0.039). Ordinal logistic regression model was applied to assess the influence of sex and age on *T. gondii*‐specific antibodies. The results demonstrated that both sex and age were significantly associated with the level of antibodies. Female tigers exhibited a significantly higher likelihood of seropositive titre (OR = 20.13, *p* = 0.026), and the probability of titre increased with age (OR = 1.91, *p* = 0.001).

**Conclusions:**

These findings highlight the potential risk of *T. gondii* transmission in captive tiger populations and emphasize the importance of regular surveillance and improved management practices in reducing zoonotic transmission.

## Graphical abstract

This study investigated *Toxoplasma gondii* specific antibodies in 135 captive tigers across seven Thai tiger parks using latex agglutination. Results revealed a high overall positivity rate of 42.96%. Ordinal logistic regression analysis demonstrated sex and age were significant risk factors, with females (OR = 20.13) and older tigers (OR = 1.91).

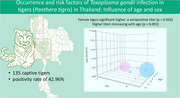

## 1 Introduction

**FIGURE 1:**
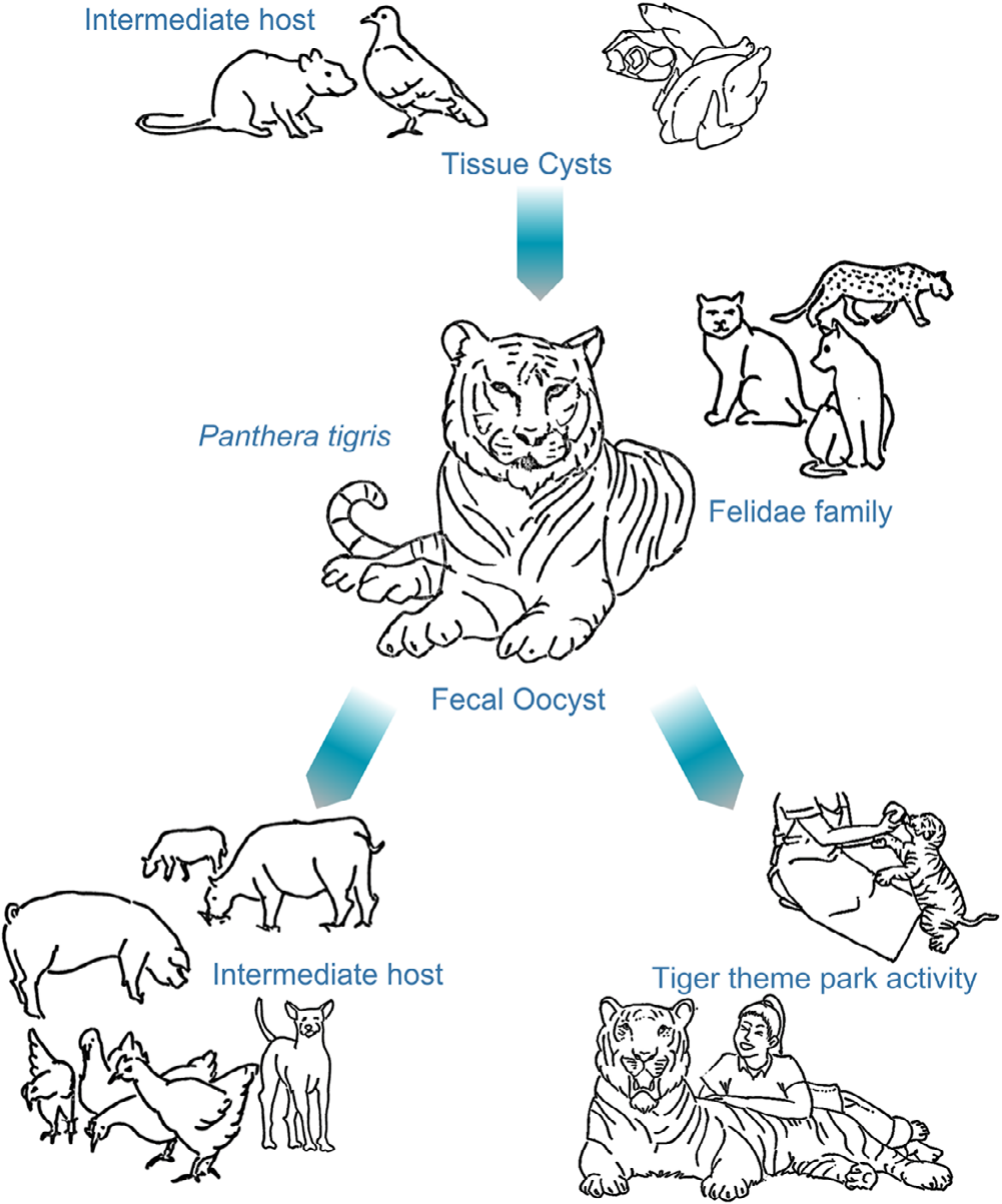
Transmission cycle of *Toxoplasma gondii* in captive tigers.

*Toxoplasma gondii* is a zoonotic protozoan parasite. It can infect a wide range of vertebrates, including birds, as intermediate hosts. Members of the Felidae family, including the tiger (*Panthera tigris*), serve as the definitive hosts (Figure 1). *T. gondii* is an apicomplexan parasite that forms protease‐resistant bradyzoite tissue cysts and uses unique mechanisms to invade host cells (Kim 2004). Felids become infected primarily through the consumption of prey containing tissue cysts with bradyzoites. The cyst wall of parasite is degraded in stomach, releasing bradyzoites and invading the intestinal epithelium (Tomasina and Francia 2020). They can also become infected by consuming food or water contaminated with sporulated oocysts. Within the intestines of the definitive host, *T. gondii* undergoes sexual reproduction, resulting in the production of oocysts. These oocysts are shed in the faeces and become infectious (sporulated) after a period in the environment. Humans and other animals can become infected by ingesting contaminated food or water containing these sporulated oocysts. (Shapiro et al. 2019). Sporulated oocysts can survive in soil for up to 1 year (Frenkel et al. 1975).

In tiger parks, tigers are typically maintained in semi‐natural conditions and are often fed raw meat as part of their dietary management. Park activities may also allow direct contact between tigers and humans, particularly in settings designed for tourism or education (Figure 1). Once infected, tigers can shed environmentally resistant oocysts in their faeces, which may contaminate enclosures, soil or other parts of the facility. This contamination poses a health risk to both other animals and humans. The presence of multiple susceptible species, combined with the ongoing use of raw meat in feeding protocols, creates an ecological environment that supports and facilitates cross‐species transmission of *T. gondii*. Most infected felids are asymptomatic. However, in immunocompromised felids, clinical signs such as diarrhoea, fever, weight loss and respiratory, ocular or neurological symptoms may appear (Zandonà et al. 2018; Moore et al. 2022). In humans, the incidence of toxoplasmosis, as well as behavioural changes associated with chronic infection, is reportedly increasing (Virus et al. 2021). Similarly, the incidence of toxoplasmic encephalitis is also rising, particularly among immunocompromised patients (Demar et al. 2012; Nardone et al. 2014). One of the main risk factors of *Toxoplasma* infection includes having several cats (Jones et al. 2009).

Diagnosis of *T. gondii* infection is commonly performed using serological assays that detect specific antibodies indicating prior exposure. As felids are the definitive hosts of *T. gondii*, serological surveillance in captive and wild felid populations is essential for understanding the epidemiology and environmental dissemination of the parasite. However, serological testing in felids, particularly large captive species, carries inherent limitations; seropositivity merely indicates past exposure and does not definitively demonstrate active infection, ongoing oocyst shedding or immediate transmission risk. Despite these limitations, previous studies report varying seroprevalence rates among felids in different settings. In European and Middle Eastern zoos, 63% of 311 small exotic felids representing 10 species were seropositive for *T. gondii* (Lücht et al. 2019). Similarly, a survey in Thai zoos and a breeding centre detected antibodies in 15.4% (21/136) of captive felids across 12 species using a commercial latex agglutination test, with titres ranging from 1:64 to 1:8192 (Thiangtum et al. 2006). In wild felids, 92% of necropsied cougars from Vancouver Island tested positive for *T. gondii* antibodies, and one individual shed oocysts, supporting the role of felids in environmental contamination associated with a major human toxoplasmosis outbreak (Aramini et al. 1998).

In this study, captive tiger samples and data from the Monitoring and Surveillance Center for Zoonotic Diseases in Wildlife and Exotic Animals (MoZWE) were analysed, aiming to identify risk factors associated with *T. gondii* infection in captive tigers, specifically evaluating the influence of sex and age on occurrence. The findings of this study may contribute to improved management and control strategies for *T. gondii* in captive tiger populations.

## 2 Materials and Methods

### 2.1 Serum Samples and Study Sites

**FIGURE 2:**
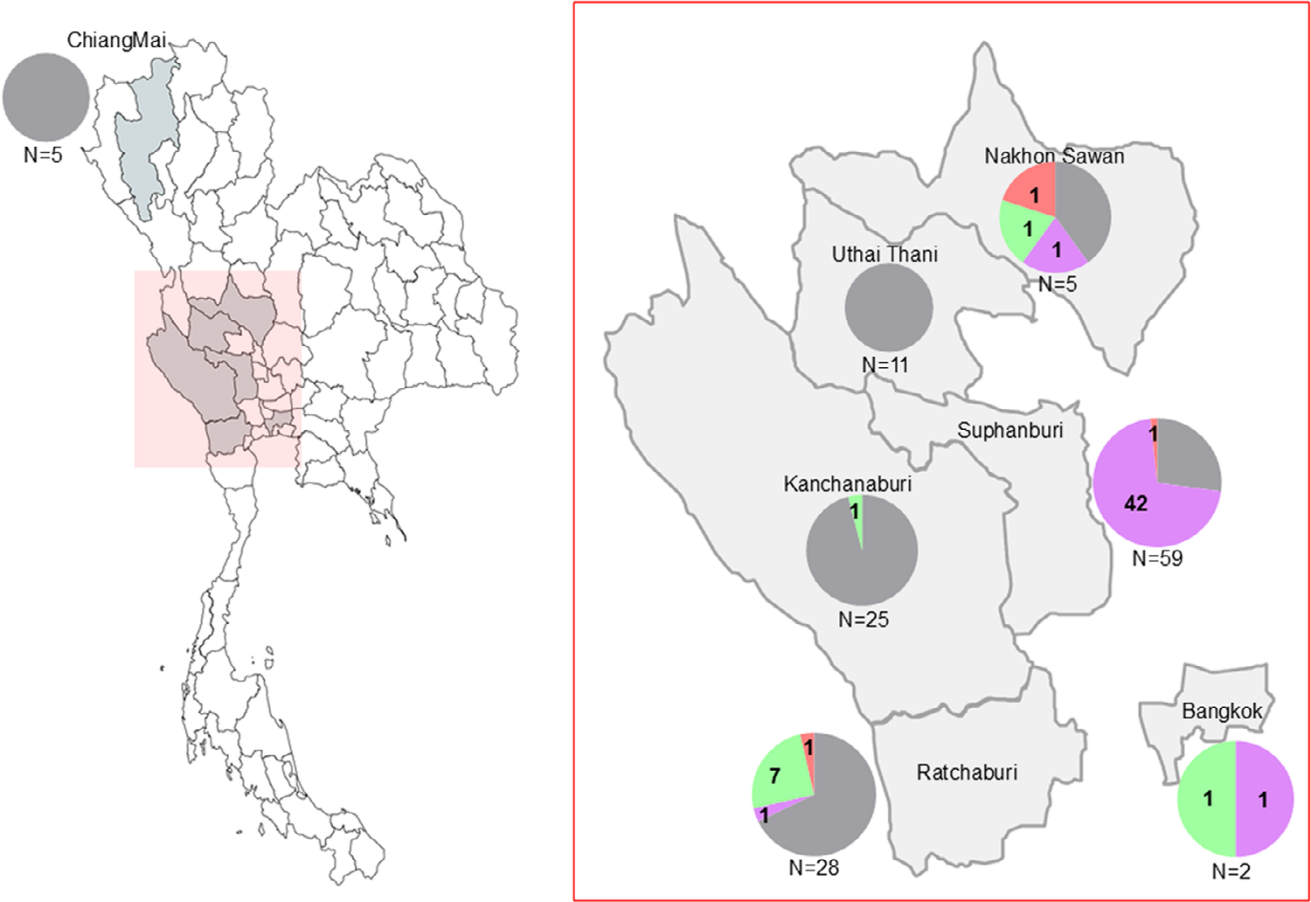
Map of Thailand showing highlighted sampling sites across seven provinces where tiger serum samples were collected with coloured dots indicating both the number of seropositive individuals and antibody titre levels. Coloured dots represent *Toxoplasma gondii* seropositivity levels: purple for low, green for moderate and red for high antibody titres.

Tiger serum samples were obtained through the MoZWE, Faculty of Veterinary Science, Mahidol University. The samples originated from 135 tigers housed in 7 tiger parks located across 7 provinces in Thailand. Six provinces were in the central region (Nakhon Sawan, Ratchaburi, Suphan Buri, Bangkok, Kanchanaburi and Uthai Thani), while one province was in the northern region (Chiang Mai) shown in Figure 2. Sex‐specific infection rates were assessed in all 135 individuals, while age‐specific infection rates were evaluated in 40 tigers for which birth date information was available.

### 2.2 Detection of *T. gondii* Antibodies Using a Latex Agglutination Assay

The latex agglutination test, LAT (Toxocheck‐MT; Eiken Chemical Company, Tanabe, Tokyo, Japan) was employed for the qualitative and semi‐quantitative detection of antibodies against *T. gondii*. This assay has demonstrated high sensitivity (97%–100%) and specificity (91.3%–98.3%) in various animal species, including members of the family Felidae (Murata 1989; Kongcharoen et al. 2015; Bártová et al. 2018). Briefly, test serum samples were initially diluted 1:8 in buffer solution. Two‐fold serial dilutions of diluted samples and positive control were performed with U‐shaped 96‐well plate to prepare 1:16–1: 2048 dilutions. Diluent buffer was used as negative control. All wells were homogenized with equal volume of sensitized latex suspension, incubated at room temperature for 12 h. Level of agglutination were determined. The antibody titre was defined as the highest serum dilution showing significant agglutination (> 1+) according to the manufacturer's instructions. According to the manufacturer's instructions, antibody titres of 1:32–1:64 were categorized as low antibody levels, 1:128–1:256 as moderate antibody levels and ≥ 1:512 as high antibody levels.

### 2.3 Statistical Analysis

The seropositive proportion of *T. gondii* was reported with a 95% confidence interval (CI) using the Clopper–Pearson score interval. Pearson's *χ* ^2^ test was applied to compare seropositive proportion across sex groups. Seropositivity level proportions were further stratified by sex (male and female) to compare group‐specific proportion. Antibody titres were categorized into three levels: low, moderate and high. Associations between sex, age and *T. gondii* seropositivity were assessed using an ordinal logistic regression model. Sex was included as a categorical variable, while age was treated as a continuous variable. Model fit was evaluated using Nagelkerke's *R* ^2^. All statistical analyses were performed using IBM SPSS Statistics version 29 (IBM Corp., Armonk, NY, USA).

## 3 Results

A total of 135 serum samples from captive tigers were analysed for *T. gondii* antibodies. The overall positive rate was 42.96% (58/135; 95% CI: 34.48–51.76). Occurrence of *T. gondii* antibodies varied across sampling sites. Positive cases were detected at five of seven sampling sites, while the remaining two sites showed no seropositivity (Table 1). The five sites with positive cases varied in the number of seropositive tigers and in antibody titres. All seropositive sites showed moderate to high antibody level, as well as low antibody levels (Figure 2). This spatial representation emphasizes variation in both occurrence and antibody titre across sampling locations.

**TABLE 1:** Number of tigers tested and seropositive for *Toxoplasma gondii* antibodies across seven different sites in Thailand.

Site	Number of tested	Positive test	Seroprevalence (%)	95% CI
Bangkok	2	2	100	15.81–100
Uthai Thani	11	0	0	0–28.49
Nakhon Sawan	5	3	60	14.66–94.73
Kanchanaburi	25	1	4	0.1–20.35
Ratchaburi	28	9	32.14	15.88–52.35
Suphanburi	59	43	72.88	59.73–83.64
Chiang Mai	5	0	0	0–52.18
Total	135	58	42.96	34.48–51.76

**FIGURE 3:**
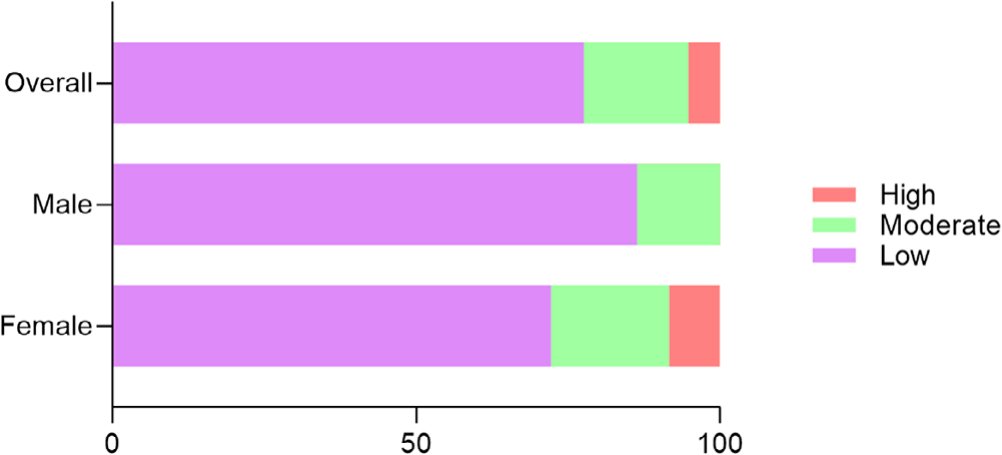
Bar chart presenting the distribution of *Toxoplasma gondii* seropositivity levels among male, female, and overall sampled tigers. Antibody titre levels were categorized as low (titres of 1:32 and 1:64), moderate (1:128 and 1:256) and high (1:512 and 1:1024). Each bar represents the proportion of tigers within each group exhibiting these antibody titre levels (low: purple, moderate: green, high: red).

When stratified by sex, antibody reaction was higher in females (51.43%, 36/70; 39.17–63.56) compared to males (33.85%, 22/65; 22.57–46.65) (*p* = 0.039). The distribution of antibodies against *T*. *gondii* across the overall population and by sex is shown in Figure 3. Among the seropositive individuals, 77.59% (45/58; 64.73–87.49) exhibited low antibody levels, while 17.24% (10/58; 8.59–29.43) and 5.17% (3/58; 1.08–14.38) exhibited moderate and high antibody levels, respectively. Seropositive females showed different antibody levels: 72.22% (26/36; 54.81–85.80) had low antibody levels, 19.44% (7/36; 8.19–36.02) moderate antibody levels and 8.33% (3/36; 1.75–22.47) high antibody levels. In contrast, seropositive males, 86.36% (19/22; 65.09–97.09) exhibited low antibody levels, and 13.64% (3/22; 2.91–34.91) had moderate antibody levels; no high antibody levels were detected in this group (Figure 3).

**FIGURE 4:**
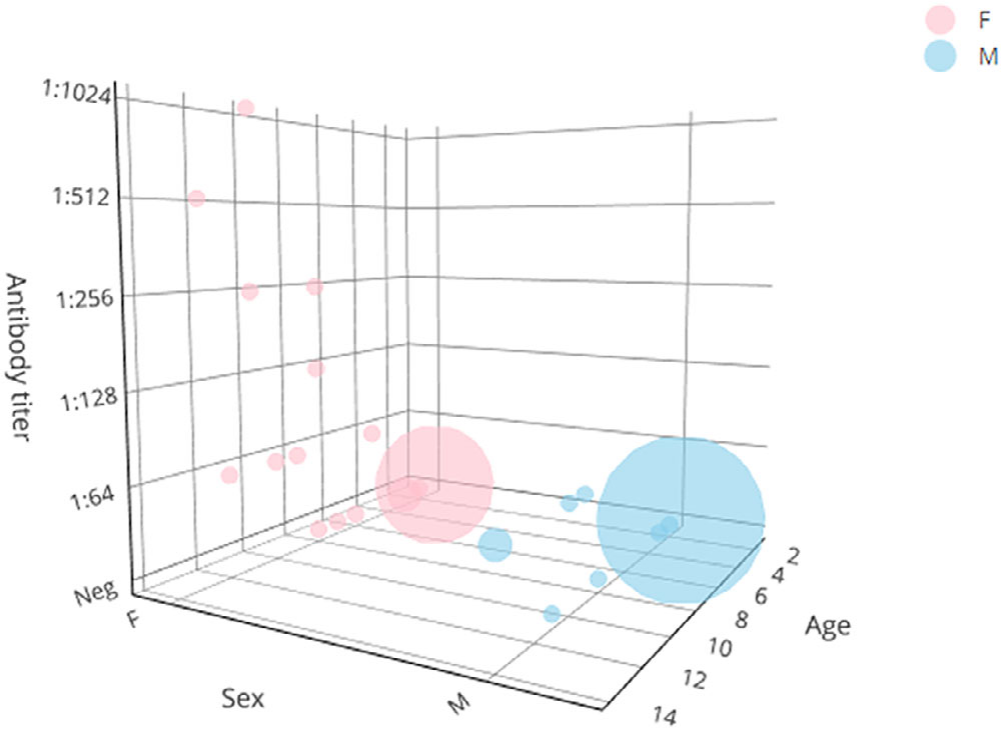
Scatter plot representing the relationship between age, sex and *Toxoplasma gondii* seropositivity levels in captive tigers.

Ordinal regression revealed that both sex and age were significantly associated with *T. gondii* seropositivity (Table 2). Female tigers had significantly higher odds of exhibiting higher antibody reactions compared to males (OR = 20.13, *p* = 0.026). In addition, increasing age was positively associated with higher seropositivity levels (OR = 1.91, *p* = 0.001) (Figure 4).

**TABLE 2:** Regression model assessing the relationship between predictor variables (age and sex) and antibody titre levels for *Toxoplasma gondii*.

Response	Predictor	*χ* ^2^	df	OR	95% CI	*p*	*R* ^2^
Antibody titre	Age	10.482		1.91	1.29–2.82	**0.001**	0.689
	Sex	4.988		20.13	1.44–280.62	**0.026**	

*Note*: Statistically significant results (*p* < 0.05) are indicated in bold.

## 4 Discussion

This study revealed a high seropositive proportion of *T. gondii* infection among captive tigers housed in facilities involving close human–animal interaction. A total of 42.96% (58/135) of the sampled tigers tested positive for *T. gondii* antibodies, with significant associations observed between seropositivity, sex and age. Female and older tigers tended to exhibit higher antibody titres. The seropositive observed in the present study is consistent with previous reports in captive members of the family Felidae from various geographical regions. Seropositivity to *T. gondii* has been documented in at least 22 felid species (Hatam‐Nahavandi et al. 2021). However, the variations in seroprevalence report across different geographical areas. In Mexican zoos, all sampled felids representing 10 species were seropositive for *T. gondii* by ELISA, with additional molecular evidence of parasitaemia and oocyst shedding (Gomez‐Rios et al. 2019). Similarly, Iatta et al. (2020) reported 100% seropositivity among tigers housed in a wildlife safari park in southern Italy. While, other regions exhibit far more moderate exposure rates, for example Lücht et al. (2019) reported a 63% seroprevalence among small exotic felids in European and Middle Eastern zoos, whereas a considerably lower rate of 15.4% was observed within Thai zoological institutions (Thiangtum et al. 2006). Collectively, these findings indicate widespread exposure to *T. gondii* among captive felids under zoological management conditions. In captive settings, exposure may be associated with husbandry related factors such as feeding raw meat, variations in feeding behaviour or food allocation, restricted enclosure areas, environmental contamination with oocysts, enclosure hygiene, water quality and contact with feral animals surrounding the facilities (Silva et al. 2007; Lücht et al. 2019). These findings emphasize the importance of routine serological surveillance, biosecurity measures and appropriate husbandry management to reduce the risk of parasite transmission in captive felid populations and minimize potential zoonotic exposure. Further studies incorporating environmental monitoring and management related risk assessment are needed to better understand transmission dynamics in captive facilities.

However, the Modified Agglutination Test (MAT) is widely recognized as the reference standard for detecting *T. gondii* antibodies. The MAT utilizes whole, formalin‐fixed *T. gondii* tachyzoites, which necessitate precise titration and carry a risk of interference (World Organisation for Animal Health [WOAH] 2018). Specifically, animal populations often contain non‐specific factors that can lead to spontaneous agglutination and subsequent false‐positive results. Conversely, interferences in animal serum have been associated with false‐negative outcomes (WOAH 2018). While, LAT is commercially and recommended reliable diagnostic by the WOAH Terrestrial Manual (WOAH 2018). The LAT utilizes polystyrene beads coated with standardized soluble *T. gondii* antigens that was ensured high reproducibility. Consequently, LAT represents a safer and practical diagnostic tool for field‐based veterinary research which requires neither specialized laboratory infrastructure nor expensive chemical reagents. The presence of IgG antibodies against *T. gondii* confirms previous exposure to the parasite, as IgG antibodies typically appear within 1–2 weeks after infection and may persist for prolonged periods (Schreiber et al. 2021). In the present study, most seropositive tigers (77.59%) exhibited low antibody levels, whereas approximately 22% showed moderate to high antibody levels. However, interpretation of antibody titres should be approached cautiously because IgM testing, IgG avidity assays and molecular confirmation were not performed. Therefore, the detected antibody levels cannot be used to distinguish recent from chronic infection or to infer active parasite proliferation. Nevertheless, serological profiling remains useful for assessing exposure patterns within captive populations and contribute to long‐term surveillance programs for captive animal health management, as antibody reactions can serve as indirect indicators of infection stage, revealing patterns of parasite transmission and identifying potential hotspots or subpopulations at elevated risk of recent or recurrent exposure (Byrne et al. 2025). The present study is limited by its cross‐sectional design, which precludes determination of infection timing. Further longitudinal investigations integrating serological approaches may provide a more comprehensive understanding of *T. gondii* epidemiology in captive tiger populations.

Significant sex‐based differences were observed in both the overall sero‐occurrence and the distribution of antibody levels. Female tigers showed a higher proportion of *T. gondii* antibodies and exhibited a broader range of antibody titres, including all observed high antibody levels. In addition, a positive association between age and seropositive was identified among female tigers, suggesting that cumulative exposure over time may contribute to increased seropositivity in older animals. Although sex‐associated differences in susceptibility to parasitic infections have been reported previously (Fischer et al. 2015). Similar associations between female sex, older age and increased *T. gondii* seropositivity have also been reported in human populations (Falusi et al. 2002; Grada et al. 2024). Nevertheless, the mechanisms underlying these differences in captive tigers remain unclear. Future studies utilizing larger cohorts and comprehensive epidemiological frameworks are needed not only to clarify these risk factors, but also to refine targeted management and biosecurity protocols for captive populations.

## 5 Conclusion

This study demonstrates a substantial seropositive proportion of *T. gondii* infection in captive tigers in Thailand. The significant associations between seropositivity, sex and age showed that female and older tigers exhibit higher antibody titres. These findings underscore the importance of targeted surveillance and management practices to reduce the risk of *T. gondii* transmission within captive tiger populations. Furthermore, this study enhances our understanding of transmission dynamics and supports the development of effective control strategies for captive wild felid populations. Strengthened biosecurity measures are essential to minimizing health risks for both animals and humans in environments where human–wildlife interactions occur.

## Author Contributions

**Phirom Prompiram**: study conception, visualization, writing – review and editing. **Chalisa Mongkolphan**: study conception, data collection, writing – review and editing. **Aeknarin Saechin**: formal analysis, visualization. **Siriporn Tangsudjai**: study conception and design, methodology, writing – original draft. **Kanaporn Poltep**: study conception and design, validation, formal analysis, interpretation of results, writing – original draft. All authors reviewed the results and approved the final version of the manuscript.

## Funding

This work was supported by the Faculty of Veterinary Science, Mahidol University.

## Ethics Statement

This study was conducted with the animal ethics approval of the Faculty of Veterinary Science at Mahidol University (MUVS‐ 2025‐07‐43).

## Consent

The authors have nothing to report.

## Conflicts of Interest

The authors declare no conflicts of interest.

## Data Availability

Additional inquiries can be directed to the corresponding author.
